# Clinical assessment of an AI tool for measuring biventricular parameters on cardiac MR

**DOI:** 10.3389/fcvm.2024.1279298

**Published:** 2024-02-05

**Authors:** Mahan Salehi, Ahmed Maiter, Scarlett Strickland, Ziad Aldabbagh, Kavita Karunasaagarar, Richard Thomas, Tristan Lopez-Dee, Dave Capener, Krit Dwivedi, Michael Sharkey, Pete Metherall, Rob van der Geest, Samer Alabed, Andrew J. Swift

**Affiliations:** ^1^ Department of Radiology, Sheffield Teaching Hospitals NHS Foundation Trust, Sheffield, United Kingdom; ^2^ Department of Infection, Immunity and Cardiovascular Disease, University of Sheffield, Sheffield, United Kingdom; ^3^ Department of Cardiovascular Disease, NIHR Sheffield Biomedical Research Centre, Sheffield, United Kingdom; ^4^ Division of Image Processing, Department of Radiology, Leiden University Medical Center, Leiden, Netherlands

**Keywords:** cardiac, magnetic resonance imaging, artificial intelligence, segmentation, time-saving

## Abstract

**Introduction:**

Cardiac magnetic resonance (CMR) is of diagnostic and prognostic value in a range of cardiopulmonary conditions. Current methods for evaluating CMR studies are laborious and time-consuming, contributing to delays for patients. As the demand for CMR increases, there is a growing need to automate this process. The application of artificial intelligence (AI) to CMR is promising, but the evaluation of these tools in clinical practice has been limited. This study assessed the clinical viability of an automatic tool for measuring cardiac volumes on CMR.

**Methods:**

Consecutive patients who underwent CMR for any indication between January 2022 and October 2022 at a single tertiary centre were included prospectively. For each case, short-axis CMR images were segmented by the AI tool and manually to yield volume, mass and ejection fraction measurements for both ventricles. Automated and manual measurements were compared for agreement and the quality of the automated contours was assessed visually by cardiac radiologists.

**Results:**

462 CMR studies were included. No statistically significant difference was demonstrated between any automated and manual measurements (*p* > 0.05; independent *T*-test). Intraclass correlation coefficient and Bland-Altman analysis showed excellent agreement across all metrics (ICC > 0.85). The automated contours were evaluated visually in 251 cases, with agreement or minor disagreement in 229 cases (91.2%) and failed segmentation in only a single case (0.4%). The AI tool was able to provide automated contours in under 90 s.

**Conclusions:**

Automated segmentation of both ventricles on CMR by an automatic tool shows excellent agreement with manual segmentation performed by CMR experts in a retrospective real-world clinical cohort. Implementation of the tool could improve the efficiency of CMR reporting and reduce delays between imaging and diagnosis.

## Introduction

Cardiac magnetic resonance (CMR) allows detailed, non-invasive assessment of the heart and its function. CMR can yield a variety of quantitative metrics - such as tissue characterization (including scar assessment) as well as quantitative assessment (chamber volumes, function and myocardial wall thickness - that have diagnostic and prognostic value in the acute and outpatient settings for several cardiopulmonary diseases, including ischemic evaluation, cardiomyopathy, myocarditis, and pulmonary hypertension (1–5).

The demand for CMR is increasing, and this is reflected in the growing inclusion of CMR in European Society of Cardiology guidelines, including those for coronary artery disease and heart failure (6, 7). There has been a rapid increase in the number of clinical CMR studies and demand is increasing 15%/year (8). In the UK, 114,967 scans are performed yearly as part of routine clinical practice (8). Locally, our tertiary UK centre has seen a continuous increase in the number of CMR studies being performed, with over 2,000 scans now performed in 2023 compared to just under 400 in 2008. Currently, each scan is evaluated manually by a consultant cardiac radiologist or other expert CMR reporter. In order to obtain cardiac volumetric and functional measurements, contours must be drawn manually around each of the cardiac chambers, a process that is time-consuming, repetitive and prone to inter-observer error.

The growth in demand for CMR is occurring in an era of worsening reporter shortages (9). The implications are reporting backlogs and delayed scan reporting, resulting in delays to patient diagnosis and management. The number of CMR consultants is estimated to be 5.4 per million/population in England, while the prevalence of cardiac disease is rising each year (8–10). The average waiting time for outpatient referrals was 41 days across England in 2018, which is considerably higher since the COVID-19 pandemic. According to the Royal College of Radiologists, it would take an additional 406 consultants employed overnight to be able to clear a 6 week backlog of scans in one month (8, 11, 12). Artificial intelligence (AI) solutions are being proposed to help alleviate the mismatch between demand and reporting capacity, with the NHS now actively funding AI solutions in this area (13).

Recent years have seen the emergence of AI tools that aim to automate repetitive and labour-intensive aspects of CMR evaluation. AI tools offer potential benefits including improved efficiency and reproducibility of contouring and segmentation (14, 15). While these offer an attractive solution to the rising demand for CMR and have demonstrated excellent results in non-clinical testing, the clinical deployment of these tools and their performance in “real world” situations remains poorly understood (16). This study aimed to assess the utility of a locally developed AI tool automated measurement of CMR volumes.

## Methods

### CMR study selection

CMR studies performed for any indication at our tertiary centre between January and October 2022 were eligible for inclusion prospectively if both manual and automated CMR measurements were available.

### Manual evaluation

**Figure 1:**
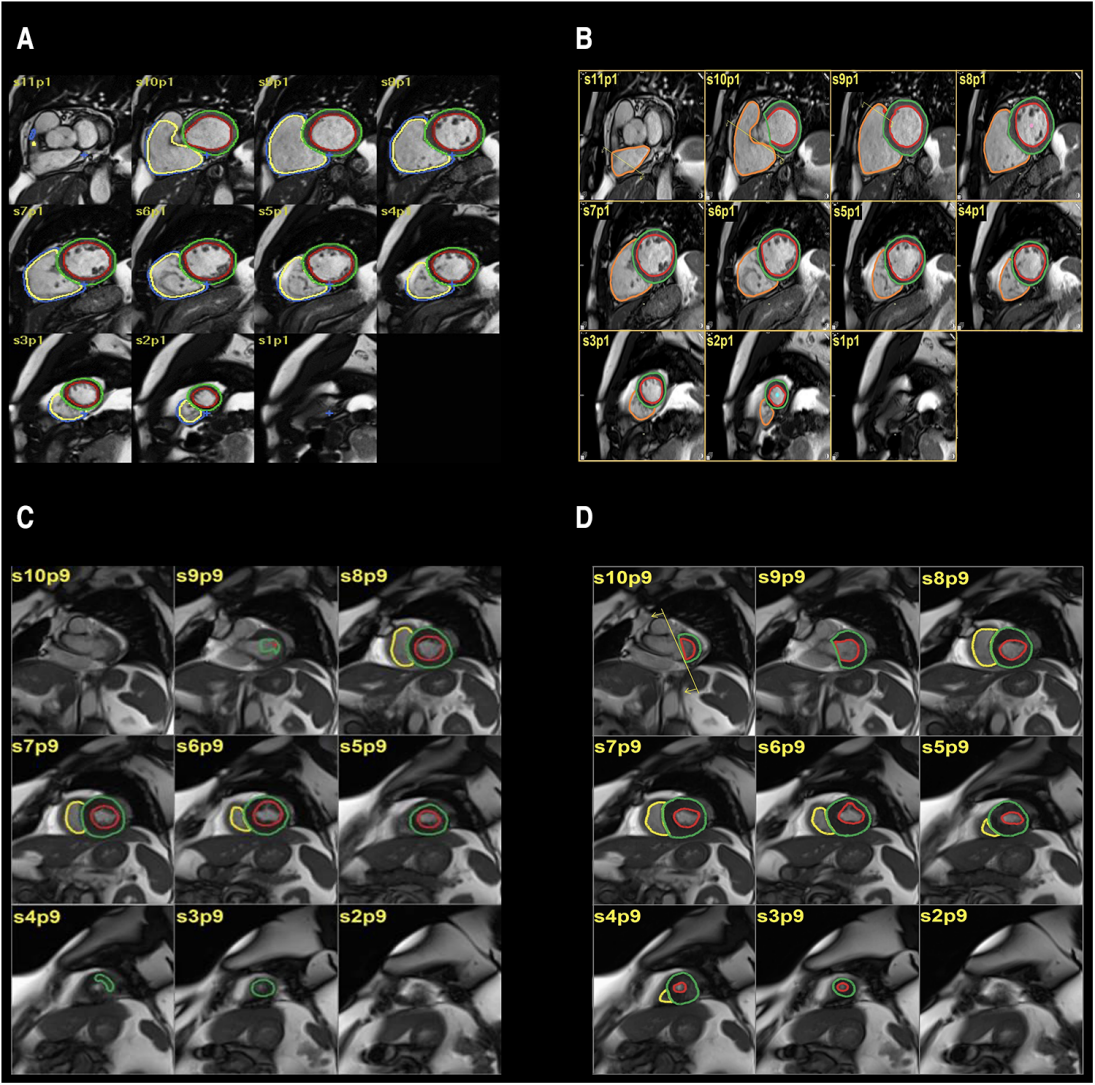
(**A,B**) automatic segmentation (**A**) and manual segmentation (**B**) of left ventricular volume showing good agreement. (**C,D**) automatic segmentation (**C**), manual segmentation (**D**) of left ventricular volume showing moderate disagreement.

Manual segmentation was performed for each included CMR study by two CMR radiographers (RT and TLD). Manual segmentation is performed at a later date to the study using the Siemens Syngo.via that does not contain a deep learning segmentation algorithm, these segmentations were drawn by the radiographers (Figure 1). Short-axis stack images were used, with epicardial and endocardial contours of the left ventricle and endocardial contours of the right ventricle for end systolic and end diastolic phase. Trabeculations were included in the blood pool and the outflow tract was included for both ventricles. The following measurements were obtained for each included CMR study: end-diastolic volume (EDV), end-systolic volume (ESV), systolic volume (SV), myocardial mass and ejection fraction (EF).

### Evaluation by AI tool

Following the acquisition of each included CMR study, the images were automatically evaluated by the AI tool. The development and testing of the tool is described in detail elsewhere (17)**.** In short, the tool is based on a convolutional neural network comprising a UNET-like architecture with 16 convolutional layers. The tool was trained using a total of 611 CMR studies from 539 patients with various cardiac abnormalities from Sheffield Teaching Hospitals NHS Foundation Trust (Sheffield, UK) and Semmelweis University (Budapest, Hungary). Testing was performed using CMR studies from a total of 4047 patients from the same centres and an external dataset from 32 centres across the UK. For each included CMR study, the AI tool automatically performed segmentation of both ventricles and provided automated measurements of EDV, ESV, SV, myocardial mass and EF across the full cardiac cycle.

Two consultant radiologists (AJS and KK) with 12 and 15 years specialist cardiac imaging experience visually assessed the automatic contours prospectively and rated them on the following disagreement scale; none, minor, moderate, and major. Those rated as minor were considered to have volume differences that were not clinically relevant, whereas the moderate and major groups represented errors that may affect the volume results.

### Data collection and analyses

**Figure 2:**
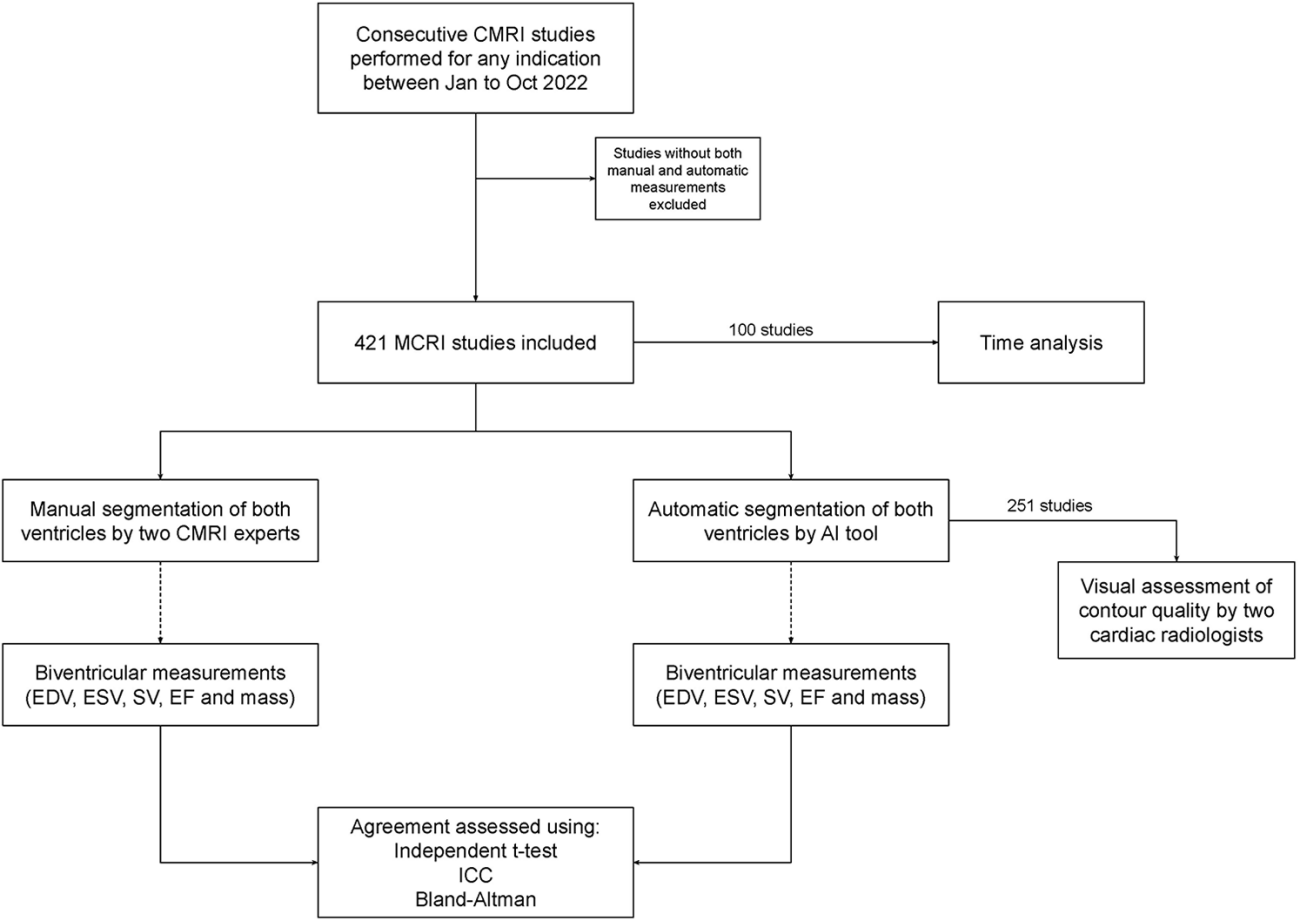
Study design and proportion of studies for each analysis

The clinical indication and final CMR diagnosis were reviewed for included studies retrospectively. These were obtained from radiological reports and information provided by clinicians for the scan. All of the scans were reported by cardiothoracic radiology consultants working in the centre. Additionally, the time taken for manual segmentation was measured prospectively on a subset of cases (Figure 2). The time for manual segmentation includes the entire process, from loading images, selecting relevant slices, the segmentation process itself and finalising contours to produce the measurements.

Statistical analysis and graph production were performed using RStudio (2022.07.1 running R 4.2.1.) and Prism (version 9.4.1; San Diego, CA, USA). Continuous data were compared using the paired T test, and categorical data compared using the chi-squared test, with a significance threshold of *p* < 0.05. Agreement between automated and manual measurements was assessed using the intraclass correlation coefficient (ICC), with values interpreted according to established thresholds: <0.6 poor, 0.6–0.8 good, >0.8 excellent (18). Bias between the measurements was also assessed using Bland-Altman analysis; these results are presented in accordance with published guidelines (19). The following measurements were compared for both right and left ventricles: EDV, ESV, SV, EF and mass.

## Results

### Included cases

462 CMR studies from 462 consecutive patients were included in the study (62.0% male, median age 57 years). Most patients were white British (68.0%); however, ethnicity information was not available for 19% of patients.

**Figure 3:**
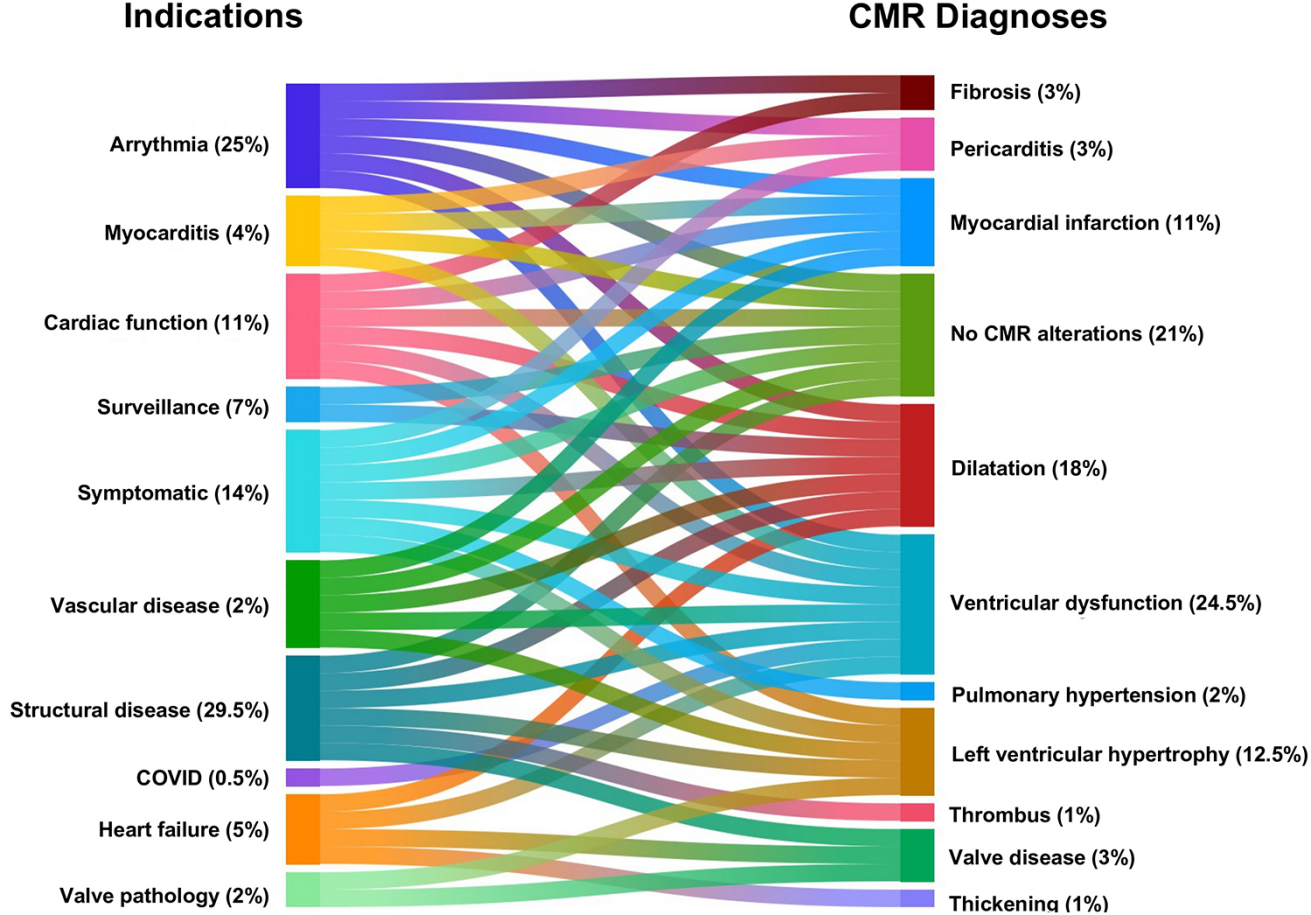
A Sankey diagram outlining the flow between indication (left) and CMR diagnoses (right).

The indications for undergoing CMR included: known structural disease (29.5%) such as dilated cardiomyopathy, hypertrophic cardiomyopathy, arrhythmogenic right ventricular cardiomyopathy (ARVC), hypertrophy or outflow obstruction; arrhythmia (25.0%), such as ventricular fibrillation, ventricular tachycardia, atrial fibrillation or ectopics; symptomatic patients (14.0%) with chest pain, shortness of breath or syncope; evaluation of cardiac function (11.0%); surveillance imaging (7.0%) for conditions such as Tetralogy of Fallot or aortic aneurysm; heart failure (5.0%), myocarditis (4.0%), valve disease (2.0%), vascular disease (2.0%), and COVID (0.5%). The CMR diagnoses included: ventricular dysfunction (24.5%); followed by no CMR alterations (21.0%); dilatation (ventricles, atria or vessels) (18.0%); left ventricular hypertrophy (12.5%); myocardial infarction (11.0%); fibrosis (3.0%); pericarditis (3.0%); valve disease (3.0%); pulmonary hypertension (2.0%); thickening (1.0%), thrombus (1.0%) (Figure 3).

### Comparison between automated and manual measurements

**Figure 4:**
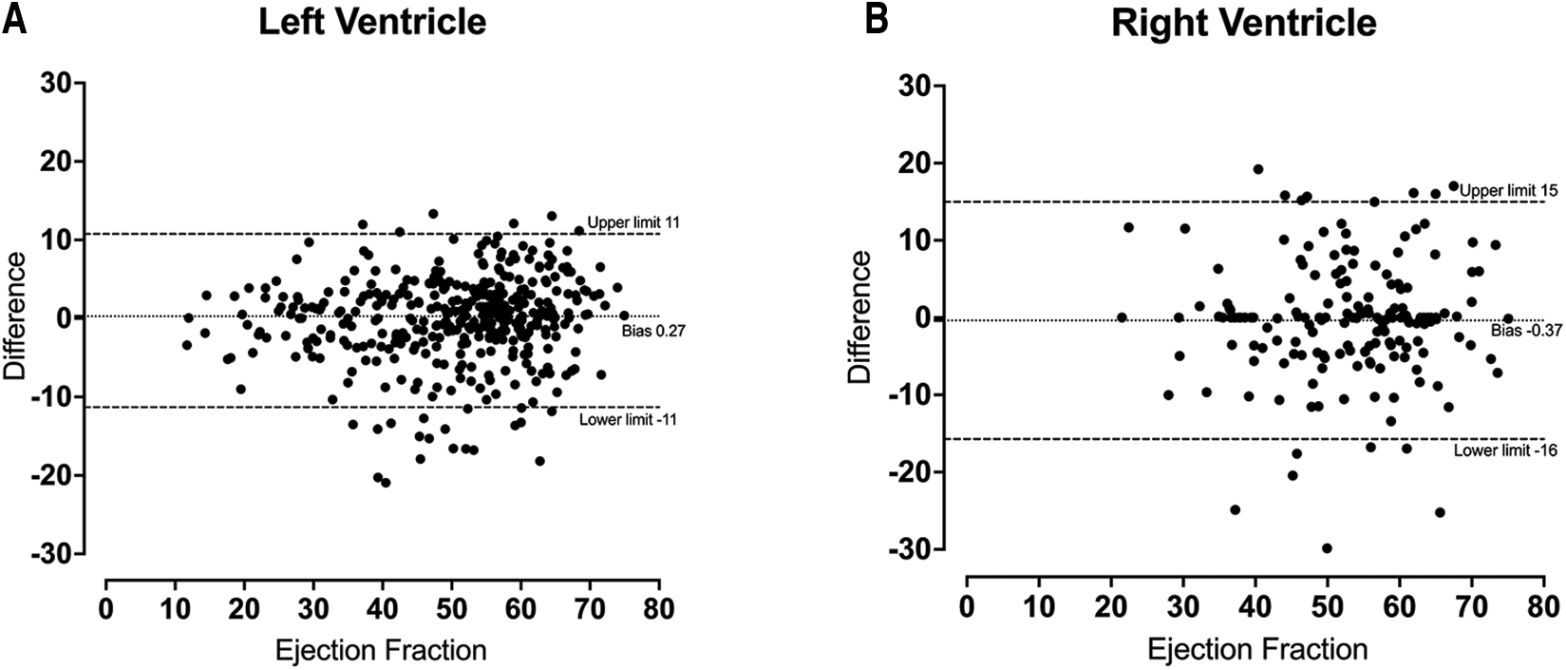
Bland-Altmann plot for left ventricular ejection fraction (**A**) and right ventricular ejection fraction (**B**) demonstrating a small amount of bias between automatic and manual measurements.

No significant difference was identified between the distribution of manual and automated ventricular measurements (Table 1). There was excellent agreement between the automated and manual measurements, with ICC values exceeding 0.85 in all cases (Table 2) and minimal bias on Bland-Altman analysis (Table 3, Figure 4).

**Table 1:** Mean and standard deviation (SD) for left and right ventricular end-diastolic volume (EDV), end-systolic volume (ESV), systolic volume (SV) in millilitres, ejection fraction (EF) and mass in grams.

Measurement	Left ventricle (*n* = 386)			Right ventricle (*n* = 126)		
	Manual	Automatic	*P*	Manual	Automatic	*P*
	(mean ± SD)	(mean ± SD)		(mean ± SD)	(mean ± SD)	
EDV (ml)	185 ± 66	197 ± 68	0.17	166 ± 57	179 ± 57	0.35
ESV (ml)	98 ± 59	104 ± 61	0.22	80 ± 37	85 ± 38	0.18
SV (ml)	88 ± 25	93 ± 26	0.19	85 ± 27	94 ± 32	0.31
EF	50 ± 14	50 ± 13	0.77	53 ± 12	53 ± 11	0.77
Mass (g)	143 ± 63	133 ± 47	0.13			

**Table 2:** Intraclass correlation coefficient (ICC) for left and right ventricular end-diastolic volume (EDV), end-systolic volume (ESV), systolic volume (SV) in millilitres, ejection fraction (EF) and mass in grams.

	Intraclass correlation coefficient			
Measurement	Left ventricle (*n* = 386)	95% CI	Right ventricle (*n* = 126)	95% CI
EDV (ml)	0.98	0.97–0.98	0.91	0.88–0.93
ESV (ml)	0.98	0.98–0.99	0.93	0.90–0.94
SV (ml)	0.93	0.92–0.94	0.87	0.82–0.90
EF	0.95	0.94–0.96	0.87	0.82–0.90
Mass (g)	0.85	0.81–0.88		

**Table 3:** Bias and standard deviation (SD) with 95% confidence interval (CI) for left and right ventricular end-diastolic volume (EDV), end-systolic volume (ESV), systolic volume (SV) in millilitres, ejection fraction (EF) and mass in grams.

Measurement	Left ventricle (*n* = 386)		Right ventricle (*n* = 126)	
	Bias (± SD)	95% CI	Bias (± SD)	95% CI
EDV (ml)	−11 ± 20	−51–28	−13 ± 32	−76–50
ESV (ml)	−5.3 ± 15	−35–24	−5.6 ± 20	−44–33
SV (ml)	−5.8 ± 13	−31–20	−8.6 ± 20	−48–31
EF	0.27 ± 5.6	−11–11	−0.37 ± 7.8	−16–15
Mass (g)	9.9 ± 40	−68–88		

### Radiologist assessment of automated contours

**Figure 5:**
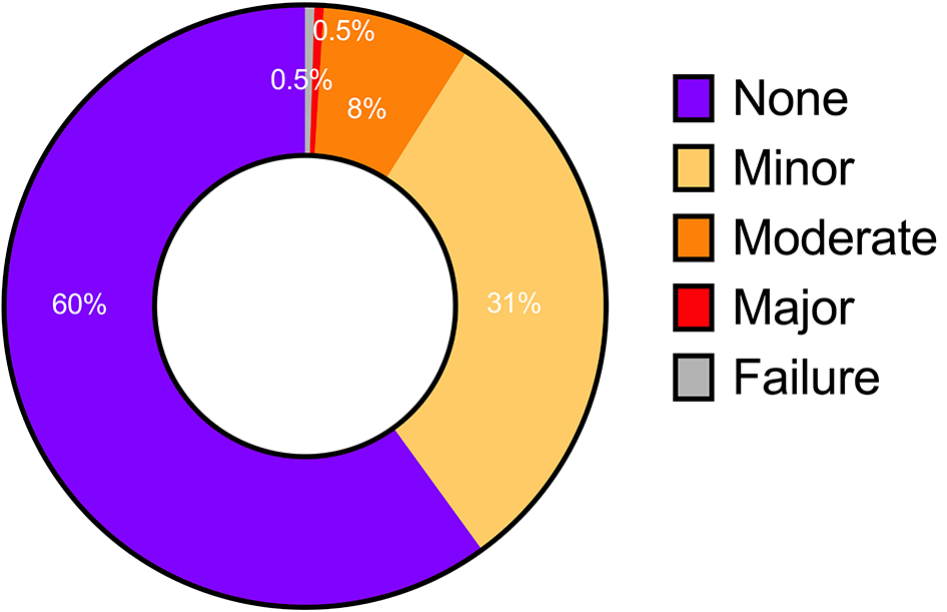
Level of agreement based on visual assessment of automatic contours. None and minor—no clinically relevant difference; moderate and major—clinically significant difference; failure—unable to produce any contouring.

The automated contours for 251 of the cases were assessed visually by a consultant radiologist prospectively. The radiologist agreed with the contours in 91.0% of cases, including 60.0% with no disagreement and 31.0% with minor disagreement (Figure 5). Moderate or major disagreement was reported in 8.0% and 0.5%, of cases respectively. Additionally, the model failed to produce any segmentation in a single case (0.4%). Disagreement was seen most commonly at the cardiac apex (29.0% of cases with disagreement), or at both the apex and base (14.0%).

### Time required for automated and manual segmentation

The length of time taken to create manual segmentation of the end-systolic and end-diastolic phase was available in 100 of CMR studies. The mean length of time across all scans was 29.1 min ± 13.9 (SD). Biventricular segmentation takes considerably longer than the left ventricle alone (Table 4), whilst a handful of cases also required flow segmentation.

**Table 4:** The length of time taken for manual segmentation in minutes for a sample of 100 cases. Biventricular segmentation takes considerably longer than left ventricular segmentation alone.

Segmentation	Time – minutes (mean ± SD)	Shortest – minutes	Longest – minutes
All scans (*n* = 100)	29.1 ± 13.9	5	67
Left ventricle (*n* = 47)	22.8 ± 10.9	5	60
Biventricular (*n* = 48)	34.8 ± 14.4	10	67
Ventricles and flow (*n* = 5)	36 ± 8.8	26	45

In contrast, the automatic method took 30 s for Siemens and 40 s for GE scanners to produce a biventricular segmentation for the full cardiac cycle. Data transfer, software initiation and graph production added an extra 45 s taking the total to a maximum of 1 min 25 s.

## Discussion

AI tools for automated segmentation of cardiac structures on CMR have the potential to improve clinical workflows. Here, we assessed the performance of our previously reported AI tool in a single centre consecutive cohort of 462 patients undergoing CMR for a variety of indications. For each CMR study, biventricular measurements of EDV, ESV, SV, EF and myocardial mass were obtained using both automated segmentation by the AI tool and traditional manual segmentation. We showed excellent agreement between the automated and manual measurements for all metrics, infrequent disagreement of cardiac radiologists with the automated contours, and considerably faster segmentation using the automated method. The results demonstrate non-inferiority of the AI tool to expert CMR segmentation in a heterogeneous real-world clinical cohort and highlight the potential of automated segmentation to improve the efficiency of CMR reporting.

Agreement between the measurements derived from automated and manual segmentation was excellent for all metrics in both ventricles. The high ICC values, minimal bias on Bland-Altman analysis and lack of statistically significant differences in measurements is indicative of robust results. Left ventricular measurements showed levels of agreement consistent with those observed previously (20). However, the right ventricle showed a slightly lower level of agreement, which has been observed in other studies and is thought to be due to significant variability in shape and intricate movement (21–23).

Furthermore, visual assessment of the automatic contours found infrequent radiologist disagreement, which is unsurprising given the close agreement in the measurements derived from automated and manual segmentation. Qualitative visual assessment confirmed the automatic contours to be accurate and reliable without needing further adjustments in 91% of cases. Our findings are highly similar to Bai et al. who have an agreement of 84.8 to 91.6% between automated and manual contours for mid-ventricular region (22). They also found the apex and base of the heart to be regions with the most disagreement likely due to the more complex anatomy and therefore more challenging contouring (22). Likewise, our cardiac radiologists frequently noted disagreements in the cardiac apex and base regions. Notably, our AI tool failed to perform segmentation in only a single case.

A key strength of this study was the evaluation of the AI tool's performance in a consecutive and unselected clinical cohort, with the included CMR studies encompassing a broad range of indications and radiological findings across multiple scanner systems. The evaluation of AI tools in their intended populations and settings ensures generalisability and is essential for their translation to routine clinical practice. The importance of generalisability is increasingly recognised and a number of multi-centre and multi-vendor studies have attempted to address this issue in the field of CMR (24, 25). Our findings add to the growing body of evidence that automatic CMR measurements derived from automated segmentation are both accurate and reliable compared to the existing standard of manual segmentation by CMR experts (21, 26).

Additionally, we have demonstrated a significant reduction in the time taken to perform segmentation: the automated method using the AI tool was able to perform segmentation in under 90 s, compared to around thirty minutes for a manual approach. The range of times for manual segmentation was due to the experience of the operators, the complexity of the cases and the quality of images. Cases with shorter time are when the operator has been working efficiently by loading cases simultaneously and the images were of high quality. It is important to note that whilst the manual segmentation is only performed at the end-systolic and end-diastolic phase, the automatic segmentation is throughout the whole cardiac cycle producing more accurate results (Table 5). This emphasises the potential for AI tools to improve the speed and accuracy of CMR interpretation and reporting in an era in which the demands for CMR continue to rise.

**Table 5:** Comparison of advantages and disadvantages of manual and automatic segmentation for cardiac segmentation.

	Manual	Automatic
Advantages	• Better able to complete challenging cases	• Quick and efficient • Accurate • Segmentation throughout the cardiac cycle • Biventricular segmentation for all cases
Disadvantages	• Time consuming • Prone to inter and intra observer variability • Segmentation typically only at end diastolic and end systolic phase • Usually, only LV unless otherwise indicated	• Performance depends on nature of training data • Can fail on challenging cases not represented in the training dataset.

There is also the wider issue of AI trustworthiness which is prevalent within cardiovascular imaging. Our model has some mitigating features as highlighted by Szabo et al. (27) such as the inclusion of multicentre data in the initial training and creating results that are explainable. This study also adds to this by demonstrating that it performs consistently in a real-world patient population without any exhibition of bias.

Our study is not without its limitations. Right ventricular segmentation is not routinely performed manually due to the additional time required - consequently, only left ventricular measurements were available for analysis in some of the included CMR studies. The AI-derived contours were assessed visually and qualitatively, with no direct quantitative comparison using a similarity score. Manual segmentations were also only performed by two experts at our centre, and it is unknown how these would vary if performed by other experts from different centres. Another limitation is the lack of comparison with commercially available algorithms provided by lenders such as Siemens and Medis. Future studies could build on our findings by examining the algorithm in a multicentre setting where more experts provide manual measurements allowing for the assessment of interobserver variability between them. Additionally, the algorithm could be compared to commercially available solutions in terms of both performance and time-saving benefits.

## Conclusion

We evaluated an AI tool for the automated segmentation of both ventricles on CMR in a consecutive single-centre real-world clinical cohort. Excellent agreement was demonstrated between measurements derived from automated and manual segmentation using multiple statistical methods. Additionally, cardiac radiologists agreed with the AI-derived ventricular contours in the majority of cases. As expected, automated segmentation was performed considerably faster than the existing standard of manual segmentation. The findings suggest that the AI tool can undertake ventricular segmentation accurately and reliably, and has the potential to improve the efficiency of CMR reporting.

## Data Availability

The raw data supporting the conclusions of this article will be made available by the authors, without undue reservation.
